# The Qure study: Q fever fatigue syndrome – response to treatment; a randomized placebo-controlled trial

**DOI:** 10.1186/1471-2334-13-157

**Published:** 2013-03-27

**Authors:** Stephan P Keijmel, Corine E Delsing, Tom Sprong, Gijs Bleijenberg, Jos WM van der Meer, Hans Knoop, Chantal P Bleeker-Rovers

**Affiliations:** 1 Radboud Expertise Centre for Q fever, Department of Internal Medicine, Division of Infectious Diseases, Radboud University Nijmegen Medical Centre, P.O. Box 9101, 6500, Nijmegen, HB, the Netherlands; 2 Department of Internal Medicine and division of Infectious Diseases, Radboud University Nijmegen Medical Centre, P.O. Box 9101, 6500, Nijmegen, HB, the Netherlands; 3 Nijmegen Institute for Infection, Inflammation and Immunity (N4i), Radboud University Nijmegen Medical Centre, P.O. Box 9101, 6500, Nijmegen, HB, the Netherlands; 4 Department of Internal Medicine, Medical Spectrum Twente, P.O. Box 50000, 7500, Enschede, KA, the Netherlands; 5 Department of Internal Medicine and division of Infectious Diseases, Canisius Wilhelmina Hospital, P.O. Box 9015, 6500, Nijmegen, GS, the Netherlands; 6 Expert Centre for Chronic Fatigue, Radboud University Nijmegen Medical Centre, P.O. Box 9101, 6500, Nijmegen, HB, the Netherlands

**Keywords:** *Coxiella burnetii*, Q fever, Q fever fatigue syndrome, QFS, Study protocol, Treatment, Doxycycline, Cognitive behavioral therapy, Placebo

## Abstract

**Background:**

Q fever is a zoonosis that is present in many countries. Q fever fatigue syndrome (QFS) is one of the most frequent sequelae after an acute Q fever infection. QFS is characterized by persistent fatigue following an acute Q fever infection, leading to substantial morbidity and a high socio-economic burden. The occurrence of QFS is well-documented, and has been described in many countries over the past decades. However, a treatment with proven efficacy is not available. Only a few uncontrolled studies have tested the efficacy of treatment with antibiotics on QFS. These studies suggest a positive effect of long-term treatment with a tetracycline on performance state; however, no randomized controlled trials have been performed. Cognitive behavioral therapy (CBT) has been proven to be an effective treatment modality for chronic fatigue in other diseases, but has not yet been tested in QFS. Therefore, we designed a trial to assess the efficacy of long-term treatment with the tetracycline doxycycline and CBT in patients with QFS.

**Methods/design:**

A randomized placebo-controlled trial will be conducted. One-hundred-eighty adult patients diagnosed with QFS will be recruited and randomized between one of three groups: CBT, long-term doxycycline or placebo. First, participants will be randomized between CBT and medication (ratio 1:2). A second double-blinded randomization between doxycycline and placebo (ratio 1:1) will be performed in the medication condition. Each group will be treated for six months. Outcome measures will be assessed at baseline and post intervention. The primary outcome measure is fatigue severity. Secondary outcome measures are functional impairment, level of psychological distress, and *Coxiella burnetii* PCR and serology.

**Discussion:**

The Qure study is the first randomized placebo-controlled trial, which evaluates the efficacy of long-term doxycycline and of cognitive behavioral therapy in patients with QFS. The results of this study will provide knowledge about evidence-based treatment options for adult patients with QFS.

**Trial registration:**

ClinicalTrials.gov: http://NCT01318356, and Netherlands Trial Register: NTR2797

## Background

Q fever, a zoonosis caused by *Coxiella burnetii*, has been present all over the world for many years [1]. Between 2007 and 2010, the south-eastern part of the Netherlands has faced the largest outbreak of Q fever ever reported. To date, more than 4000 people have developed symptomatic disease [2], and at least up to 44,000 are estimated to have been infected [3,4]. In recent years, several studies have described the sequelae of Q fever. Acute Q fever is followed by a chronic infection in 1-5% of cases [5-7]. In addition, following acute Q fever, patients frequently report long-lasting fatigue, which often persists for more than six months [8-10]. After an outbreak of Q fever in the UK, 10 years of follow-up revealed a high percentage of persisting fatigue, with almost 20% of patients fulfilling the Centre for Disease Control (CDC) criteria of chronic fatigue syndrome, compared to 4% in healthy controls [11]. A study among abattoir employees in Australia showed that 28% of patients with proven acute Q fever fulfilled the CDC criteria of chronic fatigue syndrome five years after the infection compared to none of the seronegative controls [10]. A recent study carried out in the Netherlands among 85 patients with acute Q fever found that 59% of patients had persistent symptoms at six months after disease onset, with fatigue being the most prevalent complaint in 52% of patients. Furthermore, over 25% still had complaints after one year [12]. Another recent survey in the Netherlands among 515 patients with Q fever found that 20% had severe fatigue and an impaired health status at 12–26 months of follow-up [13]. This fatigue following acute Q fever, sometimes accompanied by several other complaints, has been designated Q fever fatigue syndrome (QFS) [14-16]. According to the recently published Dutch algorithm on QFS [14], the diagnosis of QFS can be made after a uniform diagnostic work-up. There has to be a severe fatigue, which lasts for at least six months and has a reference to an acute Q fever infection. There must be an absence of fatigue before the episode of acute Q fever or a significant increase in fatigue since the acute Q fever infection. Furthermore, it is causing significant disabilities in daily practice. Finally, chronic Q fever and other causes of fatigue, somatic or psychiatric, need to be excluded.

In the Netherlands, QFS resulted in a large incurred loss due to loss of quality of life and health-related absenteeism in the past few years [17]. Currently, extrapolating the present data, at least 800 patients suffer from QFS in the Netherlands. It is expected that Q fever will remain an endemic disease, leading to a further increase in patients with QFS, stressing the need for further research into treatment regimens for QFS.

Both acute and chronic Q fever have been extensively studied in recent years; however, less attention has been given to QFS. Although QFS is a well documented finding and has already been described in 1996 [8,10], at present there is no consensus on the pathogenetic process underlying QFS [15,18,19]. In QFS, as in chronic fatigue syndrome, persistence of live microbes has been suggested [19]. Furthermore, it is still unclear whether effective treatment for QFS is possible. So far, few studies on the effect of treatment with antibiotics on fatigue after Q fever have been done. The available studies suggest a positive effect of long-term treatment with a tetracycline on performance status [20-22]; however, these studies suffer from several limitations. So far, no controlled trials have been performed and the above long-term treatment is currently not often used in clinical care of patients with QFS. Previously, it has been shown in patients with chronic fatigue syndrome (CFS) that fatigue-related cognitions and behavior can maintain chronic fatigue [23-26]. CBT for chronic fatigue is aimed at these fatigue-related cognitions and behavior thought to perpetuate the symptoms. Several systematic reviews and meta analyses demonstrated that CBT for CFS is able to reduce symptoms and to improve function in patients with CFS [26-28]. To date, the efficacy of CBT has not been studied in patients with QFS. However, our recent clinical experience with this treatment modality in a small cohort of QFS patients shows promising results.

The primary aim of our study is to determine the effect of different treatment modalities which have been suggested to be effective for patients with QFS. In this paper we describe the protocol to assess the efficacy of two treatment strategies for QFS: long-term treatment with either doxycycline or CBT.

## Methods/design

### Study design

A randomized placebo-controlled trial (RCT), the Qure study, will be performed to determine whether long-term treatment with doxycycline or CBT will lead to a reduction of fatigue and disabilities in patients with QFS. Both treatment modalities will be compared to a placebo group. This study will be performed in the Radboud University Nijmegen Medical Centre in the Q fever outpatient clinic of the department of Internal Medicine, and in the Expert Centre for Chronic Fatigue (ECCF). QFS will be diagnosed at the Q fever outpatient clinic after a uniform diagnostic work-up according to the Dutch algorithm on QFS. Once the diagnosis is established, study eligibility will be assessed by the first author (SPK) according to specific inclusion and exclusion criteria (Tables 1 and 2). Eligible patients will be asked to participate in the Qure study after receiving verbal and written information about the study. If patients are willing to participate, written informed consent will be obtained. After inclusion, an individual study code is allocated to the participants. Results from the clinical assessment before inclusion will be used as baseline measures as well. If patients decide not to participate in this study, an attempt will be made to elucidate the reason for this, but patients are not obligated to motivate their refusal.

**Table 1:** Inclusion criteria

Inclusion criteria^*^	
(1)	Males or non-pregnant, non-lactating females who are 18 years or older
(2)	Laboratory-proven acute Q fever since the year 2007 and/or positive serology fitting a past infection with *Coxiella burnetii*
(3)	AND being severely fatigued, defined by scoring ≥ 35 on the subscale fatigue severity of the CIS
(4)	AND being fatigued for at least 6 months
(5)	AND being disabled because of the fatigue, defined by scoring 450 or higher on the SIP
(6)	Subject must sign a written informed consent form

* All participants have to meet the criteria for QFS according to the recently published Dutch algorithm on QFS [14]. In addition to the mentioned inclusion criteria and according to the Dutch algorithm on QFS, there has to be a severe fatigue with a reference to an acute Q fever infection. Furthermore, there must be an absence of fatigue before the episode of acute Q fever or a significant increase in fatigue since the acute Q fever infection.

Abbreviations: CIS = Checklist Individual Strength questionnaire, SIP = Sickness Impact Profile questionnaire.

**Table 2:** Exclusion criteria

**Exclusion criteria**	
(1)	Fulfilling criteria for chronic Q fever^*^
(2)	Acute Q fever in the setting of a prosthetic cardiac valve or aneurysm surgery or stenting, necessitating prophylactic use of doxycycline
(3)	Pregnancy or unwillingness to use effective contraceptives during the entire study period
(4)	Imminent death
(5)	Inability to give informed consent
(6)	Allergy or intolerance to doxycycline
(7)	Somatic or psychiatric illness that could explain the chronic fatigue
(8)	Subjects who are currently enrolled in other investigational drug trials or receiving investigational agents
(9)	Receiving or having received antibiotics for > 4 weeks, potentially active against *Coxiella burnetii*, for any other reason since Q fever diagnosis
(10)	Subjects who are receiving and cannot discontinue barbiturates, phenytoin, or carbamazepine^**^
(11)	Moderate or severe liver disease (AF, ALT, AST > 3 times the upper limit of normal)
(12)	Current engagement in a legal procedure concerning financial benefits^#^

* According to the guideline chronic Q fever from the *Dutch Q fever consensus group*[29].

** These drugs may increase the metabolism of doxycycline; consequently, reducing the half-life of doxycycline.

# Temporary exclusion criterion, while current involvement interferes with the effectivity of cognitive behavioral therapy [30]. Once the appeal procedure ends, subjects can be included.

Abbreviations: AF = alkaline phosphatase, ALT = alanine aminotransferase, AST = aspartate aminotransferase.

### Study population

It is intended to include 180 patients diagnosed with QFS, equally randomized between three different treatment modalities, namely long-term doxycycline (n = 60), CBT (n = 60) or placebo (n = 60). All eligible patients directly referred to Radboud University Nijmegen Medical Centre will be asked to participate in this study. Patients with a suspicion of QFS presenting to other hospitals in the area will be referred to the Q fever outpatient clinic of the Radboud University Nijmegen Medical Centre for screening and enrollment in the study. In addition, all physicians working at specific Q fever outpatient clinics in other hospitals will be informed about the study. Patients connected to Q-uestion, a foundation for patients with Q fever, will be informed about the Qure study by newsletters, and a brief description will be available at the website of Q-uestion. Furthermore, patients who participated in previous studies on Q fever in the past few years (Q-Quest II study, ZonMw dossier number: 204004003, and The PrediQt study, ZonMw dossier number: 205520003, NL36477.091.11), will be informed about the Qure study by letter. Finally, all general practitioners in the endemic Q fever region will be informed about this study by letter.

### Ethical approval

According to the Dutch law, this study has been reviewed and approved by the Medical Ethical Review Committee of the Radboud University Nijmegen Medical Centre (registration number 2011/069, NL35755.091.11). This study will be conducted according to the principles of the Declaration of Helsinki. The inclusion of patients started in May 2011.

### Baseline assessment

**Figure 1:**
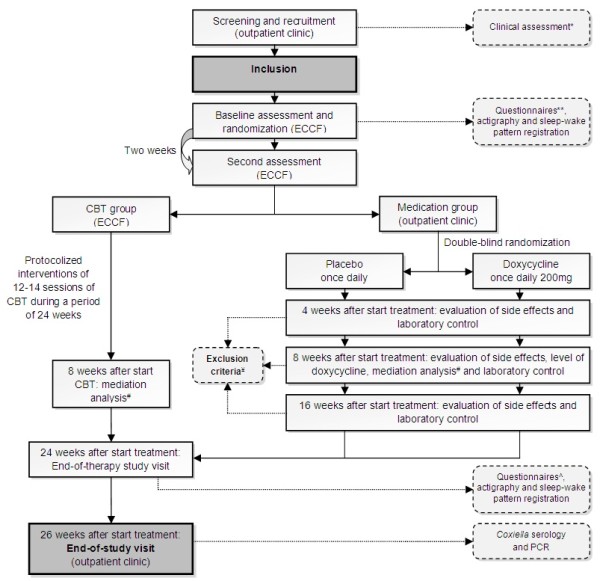
**Flowchart of trial design.** * According to the Dutch guideline Q fever fatigue syndrome [14]. Including questionnaires: general questionnaire, CIS, SIP total score. ** General questionnaire, PARS, SES28, IMQ, CBRSQ, JFCS, CAL, and SCL90. ^#^ Questionnaires used for mediation analysis: PARS, SES28, IMQ, CBRSQ, and CIS. ¥ Exclusion criteria: pregnancy; serious adverse events; AST/ALT > 5 times normal value; AF > 3 times normal value; > 10 days use of quinolon, co-trimoxazol, macroliden or tetracycline; or discontinuation of study medication > 7 consecutive days. ^ CIS, PARS, IMQ, JFCS, SIP, SES28, CBRSQ, and SCL90. Abbreviations: CIS = Checklist Individual Strength, SIP = Sickness Impact Profile, ECCF = Expert Centre for Chronic Fatigue, PARS = Physical Activity Rating, SES28 = Self Efficacy Scale, IMQ = Symptom focusing of the illness Management Questionnaire, CBRSQ = Cognitive and Behavioral Responses to Symptoms Questionnaire, JFCS = Jacobson Fatigue Catastrophising Scale, CAL = Causal Attribution List, SCL90 = Symptom Checklist 90, CBT = cognitive behavioral therapy, AST = aspartate aminotransferase, ALT = alanine aminotransferase, AF = alkaline phosphatase.

After inclusion, participants will first visit the ECCF for the baseline assessment, including questionnaires and measurement with an actometer (see Figure 1). An actometer is a motion-sensing device worn at the ankle that registers and quantifies physical activity. The actometer has a piezoelectric sensor that is sensitive in three directions. Accelerations of the built-in sensor larger than a predefined threshold are considered as activity and are stored in an internal memory every 5 minutes. It is worn day and night during a period of twelve consecutive days [31]. A general physical activity score that expressed the mean activity level over this period in the mean number of accelerations per 5 minute interval will be calculated. During the period of twelve days participants rate fatigue, pain, and activity levels on a pre-scheduled Self-Observation List four times daily on a scale of 0 (not at all) to 4 (very much).

### Randomization procedure and blinding

The randomization order is created by an independent biostatistician using block-randomization. An administrative assistant with no affiliation to the project group made envelopes for individual study codes ranging from 1–180, according to the randomization list. At the end of the first visit to the ECCF, participants receive their envelope (which contains a corresponding number coherent to the individual study code) from the psychological assistant, to see to which treatment they are randomized. First, participants will be randomized between CBT and medication (ratio 1:2). Secondly, double-blinded randomization between doxycycline treatment or placebo (ratio 1:1) will be performed within the medication condition by the study pharmacist (department of Clinical Pharmacy, Radboud University Nijmegen Medical Centre). The double-blinded randomization assignment will be known to the study pharmacist only, and is available in a sealed envelope stored at the pharmacist’s office for emergency use. If the code is broken, it will render the participant not eligible. The first randomization list and second double-blinded randomization list will be made available respectively by the independent biostatistician and the study pharmacist to the principal investigator when the entire study is completed. Obviously, allocation to the CBT intervention cannot be blinded.

### Interventions

#### Study medication

Preparation and labeling of doxycycline and placebo will be performed by the Clinical Trials Unit department of the Clinical Pharmacy of the Radboud University Nijmegen Medical Centre, and will be done according to the relevant Good Manufacturing Practice (GMP) guidelines. Study medication will be prepared as capsules with identical appearance. Participants allocated to study medication will be treated at the Q fever outpatient clinic. Participants will receive either doxycycline (200 mg once daily) or placebo (once daily), both orally administered, for a period of 24 weeks. Study medication will be provided by the first author (SPK). For safety considerations all participants in the medication condition will visit the Q fever outpatient clinic 4, 8, and 16 weeks after start of the treatment (see Figure 1). Furthermore, liver enzymes will be checked, and drug utilization will be recorded. Therefore, patients are required to bring the study medication to all visits. In addition, blood samples drawn 8 weeks after start of treatment will be stored by the study pharmacist, who performed the double-blinded randomization. Eventually, doxycycline levels will only be determined in participants receiving doxycycline, and results will be kept secret until the entire study is completed. After completion, it is known whether doxycycline levels were sufficient to sort out effect [32]. Participants will be excluded in case of: serious side effects; aspartate aminotransferase (AST)/alanine aminotransferase (ALT) levels more than 5 times the upper limit of normal; alkaline phosphatase (AF) levels more than 3 times the upper limit of normal; more than 10 days use of antibiotics potentially active against *C. burnetii* (co-trimoxazol, quinolon, macrolides or tetracyclines); or discontinuation of study medication for more than 7 consecutive days.

#### Cognitive behavioral therapy

CBT for QFS is aimed at changing the beliefs and behaviors assumed to maintain fatigue. On average, CBT consists of 12–14 sessions over a period of 24 weeks, and is individually delivered by trained cognitive-behavioral therapists from the ECCF, according to a written treatment manual. The treatment is based on CBT for CFS [33]. First, the model of fatigue perpetuating beliefs and behaviors is explained to patients. At the start of the therapy patients formulate their goals in behavioral terms. These goals usually include the resumption of work, hobbies, and other activities that imply that the patient is no longer severely fatigued and disabled, which is the goal of CBT for QFS. Patients regulate their bedtimes and stop sleeping during the day in order to stop possible disruption of the circadian rhythm. During the sessions, the therapist elicits and challenges patients’ non-accepting and catastrophising beliefs with respect to fatigue. Additionally, patients are taught how to distract their attention from their fatigue. Two groups of patients are discerned: relatively active patients, who are characterized by bursts of activity followed by periods of relative inactivity, and low active patients, who have extremely low activity levels on most days [31]. Relatively active patients first learn how to divide their activities more evenly across the day. Low active patients start with a graded activity program immediately after the initial cognitive interventions. This activity program consists of daily walking or cycling, which is gradually increased. The increase in activity is not determined by the level of symptoms, but is time contingent. When patients succeed in increasing their physical activity, they also start to increase their social and mental activities. In the last phase of therapy, patients work systematically towards reaching their goals, which are formulated at the start of the therapy. Following this, they are encouraged to perceive feelings of fatigue as a normal part of an active and healthy life.

### Post intervention

Twenty-four weeks after start of treatment, all participants visit the ECCF for the end-of-therapy study visit, including assessment of the outcome measures (see Figure 1). Twenty-six weeks after start of treatment, participants visit the Q fever outpatient clinic for the end-of- study visit. During this end-of-study visit, *C. burnetii* serology and PCR will be determined.

### Outcome measures

The primary outcome measure is the fatigue severity measured by the subscale *fatigue severity* (8 items, 7-point Likert Scale) of the Checklist Individual Strength (CIS questionnaire) [34] with a severity range from 8–56. High scores indicate a high level of fatigue. Patients with a cut-off score of ≥35 are classified as severely fatigued. This questionnaire has excellent psychometric properties, including good reliability and discriminative validity [35,36].

Secondary outcome measures are:

(1) Level of functional impairment measured with the Sickness Impact Profile (SIP) [37,38]. The SIP is an instrument that is used to gauge sickness-related dysfunction. The weighted total score on eight sub-scales of the SIP8 (SIP8 total score) will be used to assess functional disability in all domains of functioning. This instrument is reliable with sufficient content validity, and it shows good correlations with other health status and functional status measures [39].

(2) Level of psychological distress measured with the total score of the Symptom Checklist 90 (SCL90). The SCL90 consist of 90 items scored on a five-point scale. Scores range from 90–450. A low total score reflects psychological well-being. The SCL-90 is a reliable and valid instrument [40].

(3) *C. burnetii* serology (immunofluorescence assay; Focus Diagnostics, Inc., Cypress, CA, USA) and serum PCR.

Other study parameters will be: demographic data; data on symptoms, diagnosis and treatment of acute Q fever; previous history; serology results performed before inclusion in the study; use of medication, smoking, and the use of alcohol or drugs; and data on self reported symptoms, disabilities, and behavioral factors.

### Mediation analysis

Testing mediation is a strategy to identify variables that intervene in the relationship between treatment and outcome. Mediation analysis can help to better understand how treatment works [41]. To assess a change in variables that might affect fatigue severity, possible mediators and fatigue severity will be assessed at baseline, eight weeks after start of treatment, and at end of therapy in all treatment modalities (see Figure 1). The proposed mediators are fatigue related cognitions and behaviors. Four instruments will be used to assess the mediators: 1) Subscales ‘resting/avoidance’, ‘all-or-nothing’ behavior, and ‘catastrophising’ of the Cognitive Behavioral Responses to Symptoms Questionnaire (CBRSQ) [42], 2) Subscale focusing on symptoms of the Illness Management Questionnaire (IMQ) [43,44], 3) Total score on the Physical Activity Rating Scale (PARS, measuring the level of confidence and expectation on fatigue performing 16 different activities, rated on a five-point scale), and 4) Total score on the Self Efficacy Scale (SES28) [45].

### Withdrawal of individual participants

Participants are informed that they can stop participating in the study at any time, without consequences. Although participants will be asked for the reason for discontinuation, giving a reason for withdrawal is not obligatory. The investigator can decide to withdraw a participant from the study in case of medical urgency. In addition, study medication will be stopped in case of pregnancy, and the participant will be withdrawn. According to the Intention To Treat (ITT) principle the analysis will be based on the initial treatment intent. Therefore, in case of discontinuation, all efforts will be made to complete and report the observations as thoroughly as possible. A complete final evaluation in accordance to the study protocol end-of-therapy study visit will be performed if the withdrawn participant agrees. Because of absence of an evidence-based treatment for QFS, other treatment options for QFS in regular health care for withdrawn participants in the CBT group are not available. Long-term doxycycline treatment is not offered, because of possible (serious) side-effects and a lack of evidence so far.

### Adverse events

Adverse events are defined as any undesirable experience occurring to a subject during the study, whether or not considered related to the experimental treatment. All adverse events in the medication condition will be recorded during the pre-scheduled controls at the outpatient clinic, and, if applicable, during the trial if spontaneously reported by the participant. The most frequent side-effects of doxycycline include gastrointestinal complaints, like nausea and diarrhea, and photo-sensibilisation. Other side-effects are rare. The drug should not be given to children and to pregnant women. This RCT involves a non-critical indication for the use of doxycycline, and the drug under investigation is well characterised and commonly used in daily practice. Even though the delivery of CBT to adults is considered safe [46,47], all adverse events reported spontaneously by the participant or observed by the therapist will be recorded by the psychological assistant at pre-scheduled time-points during the therapy (8 weeks after the start of therapy, and 24 weeks after start of therapy). All adverse events will be followed until they have abated, or until a stable situation has been reached. If applicable, serious adverse events in both groups will be reported according to the principles of Good Clinical Practice (GCP).

### Statistical analysis

The primary analysis will be the comparison between the experimental groups (CBT or doxycycline) and the placebo group. ITT will be the basis for all analysis. The primary analysis will be done on the data of completers. Completers are all participants who completed the post intervention measurements. When statistical significant differences are found, a sensitivity analysis will be performed on the basis of different assumptions about the values of missing data. To determine if there is a significant difference between the intervention arm and placebo condition, ANCOVA will be used with the outcome measure on the second assessment as dependent measure, the baseline score as covariate, and condition as fixed factor. A priori contrasts will be defined for the factor condition comparing CBT versus placebo, and comparing doxycycline versus placebo. For the secondary outcome measures, namely psychological distress and functional limitations, the same analysis will be repeated, but with the secondary outcome measures at the second assessment as dependent variable, and the scores at baseline as covariate. In this kind of trials ANCOVA yields greater power than other statistical methods [48]. Statistical significance will be assumed at *p* < 0.05 in all analysis. Data will be presented as quantitative results.

### Power calculation

The power calculation is based on the estimated maximal number of eligible patients who will be available for the study. In the Netherlands there has been only one major outbreak of Q-fever. Since then, the number of new cases is limited. Furthermore, following the outbreak several studies investigating the symptoms following Q-fever are ongoing which limits the number of eligible patients that will be available to enter the present study. The maximal number of available patients is estimated to be 180, 60 patients for each arm of the study. We assumed a drop-out rate of 20 percent, leaving a sample size for the power calculation of 50 participants per arm. Compared to a *t*-test, using ANCOVA increases statistical power. The sample size of 50 can be divided by a design factor of 0.884 (1–0.34^2^), with 0.34 being the correlation between the CIS fatigue severity at baseline and second assessment [49]. The required effect size was estimated using G-Power 3.1.5. based on a sample size of 56, a power of 0.80 and an alpha of 0.05. The analysis showed that we need to assume a moderate controlled effect size of 0.53 to obtain a power of 0.8 for demonstrating a significant difference between the results in the treatment groups and in the placebo group.

## Discussion

The Qure study will be the first randomized placebo-controlled clinical trial to assess the efficacy of long-term treatment with doxycycline and CBT in adult patients with QFS. A limited amount of previous uncontrolled studies suggest a positive effect of long-term treatment with a tetracycline on performance state. The result of one study shows improvement in symptoms, including fatigue, in all patients after 3 months of treatment. However, not all patients met the current criteria for QFS, whereas 7 patients were PCR positive, meeting the current criteria for chronic Q fever [20]. Furthermore, patients were included with complaints lasting for only 3 months, whereas chances for spontaneous recovery are high in the first 6 months after the initial infection. The other study, primarily focussing on the role of *C. burnetii* in CFS, reports improvement in performance status, a decreased mean headache score, and a decrease in mean weekly temperature after treatment [21]. However, of the 54 patients included, 34 patients were PCR positive at baseline, suggesting chronic Q fever. Furthermore, patients were included with complaints lasting for only 1 month. Therefore, these results cannot be extrapolated, and this long-term treatment is currently not often used in clinical care of patients with QFS. Furthermore, the efficacy of CBT in patients with QFS has not been evaluated in a randomized design. Currently, the decision whether or not to treat is made arbitrarily, as evidence-based strategies are lacking. The Dutch outbreak offers us a great and maybe the only opportunity to conduct research on the best treatment of QFS.

In conclusion, the Qure study will provide greater insight into effectiveness of treatment options for adult patients with QFS. If an effective treatment modality for QFS will be found, significant benefit can be achieved in quality of life, efficiency in treatment and cost-effectiveness. Furthermore, this study will possibly contribute to the establishment of evidence-based guidelines for the treatment of QFS.

## Abbreviations

AF: Alkaline phosphatase;ALT: Alanine aminotransferase;AST: Aspartate aminotransferase;CBT: Cognitive behavioral therapy;CBRSQ: Cognitive behavioral responses to symptoms questionnaire;CDC: Centre for disease control;CFS: Chronic fatigue syndrome;CIS: Checklist individual strength;ECCF: Expert centre for chronic fatigue;GCP: Good clinical practice;GMP: Good manufacturing practice;IMQ: Illness management questionnaire;ITT: Intention to treat;PARS: Physical activity rating scale;RCT: Randomized controlled trial;SCL90: Symptom checklist 90;SES28: Self efficacy scale;SIP: Sickness impact profile (questionnaire);QFS: Q fever fatigue syndrome.

## Competing interests

The authors declare that they have no competing interests.

## Authors’ contributions

SPK participated in the design of the study and is responsible for data collection and analysis, and for drafting the manuscript. CED participated in the design of the study as an expert on infectious diseases, and will supervise the study and data collection. TS participated in the design of the study as an expert on infectious diseases. GB participated in the design of the study as an expert on chronic fatigue, helped to coordinate and supervise the study, and will be responsible for the logistics surrounding cognitive behavioral therapy. JvdM participated in the design of the study as an expert of infectious diseases and chronic fatigue, and helped to coordinate and supervise the study. HK participated in the design of the study as an expert on chronic fatigue, and will be responsible for the logistics surrounding cognitive behavioral therapy. CPBR initiated and participated in the design of the study as an expert on infectious diseases, obtained funding for the study, and will coordinate and supervise the study and data collection. All authors revised the draft manuscript and approved the final manuscript.

## Pre-publication history

The pre-publication history for this paper can be accessed here:

http://www.biomedcentral.com/1471-2334/13/157/prepub
